# Two Decades of Human Rabies in Brazil: Epidemiological Trends, Emerging Risks and Treatment Challenges

**DOI:** 10.1590/0037-8682-0110-2025

**Published:** 2025-08-01

**Authors:** Raquel Gomes Catozo, Cesar Martin Leyva Molina, Amanda Lopes da Silva, Joana Rocha da Silveira Barreto de Aguiar, Luiza Barbosa, Bruno Luiz Miranda Guedes, Mariene Ribeiro Amorim, Paulo Eduardo Brandão, Camila Malta Romano

**Affiliations:** 1 Universidade de São Paulo, Faculdade de Medicina, Laboratório de Virologia, São Paulo, São Paulo, SP, Brasil.; 2 Universidade de São Paulo, Faculdade de Medicina Veterinária e Zootecnia, Laboratório de Zoonoses Virais, São Paulo, SP, Brasil.; 3 Universidade de São Paulo, Hospital das Clínicas, São Paulo, SP, Brasil.

**Keywords:** Rabies, Zoonotic diseases, Epidemiology, Wildlife, Public health, Brazil

## Abstract

**Background:**

Rabies poses a serious public health challenge in Brazil, and its epidemiology has undergone significant changes over the past two decades. Although canine transmission of rabies has been controlled through national vaccination programs, new risks have emerged, particularly from wildlife reservoirs.

**Methods:**

We performed a retrospective analysis of human rabies cases reported in Brazil between 2001 and 2025 using epidemiological surveillance data, scientific literature, and public health reports. We focused on trends in transmission sources, changes in affected populations, and challenges in prevention and treatment, particularly regarding the availability of post-exposure prophylaxis (PEP).

**Results:**

A total of 188 human rabies cases were reported in Brazil between 2001 and 2025 (average: 7.52 cases per year). Most of the cases occurred in the northern and northeastern regions of the country. Bats were the most common source of transmission, followed by dogs and non-human primates. Although rabies is preventable, most of the cases reported within the study period resulted in death. PEP was administered in most cases; however, the treatment may have been delayed, incomplete, or initiated after the onset of symptoms, which limited its effectiveness. Only two patients survived, and both of them received intensive care and were treated using modified therapeutic protocols.

**Conclusion:**

The transition from canine- to bat-mediated rabies highlights the need for enhanced surveillance and targeted prevention strategies. Despite advances in canine rabies control, ensuring PEP accessibility and strengthening public health interventions in high-risk areas remain critical for reducing the incidence of human rabies in Brazil.

## INTRODUCTION

Rabies is a tropical zoonotic disease caused by the rabies virus (Lyssavirus rabies), which belongs to the genus Lyssavirus and the family Rhabdoviridae. This family includes a wide range of viral species with extensive host diversity, including vertebrates (mammals, fish, and reptiles), invertebrates, and plants^1^.

The three genera of viruses that cause rabies in mammals are Vesiculovirus, Lyssavirus, and Ephemerovirus. The rabies virus is the most prominent member of the Lyssavirus genus, with a global prevalence and a long research history. However, several other Lyssaviruses can also cause fatal rabies-like diseases. Eighteen rabies-related Lyssaviruses have been identified to date, and they include Mokola virus, European Bat Lyssavirus 1 and 2, and Australian Bat Lyssavirus^2^. Several cases of human lyssavirus infection associated with bats have been reported in Europe, Asia, and Australia. In continental Europe, the European bat lyssavirus 1 (EBLV-1), which is linked to the serotine bat (Eptesicus serotinus), has been implicated in at least three human infections. EBLV-2, which has been identified in the Netherlands, Switzerland, Finland, the United Kingdom, and Germany, is transmitted by Daubenton’s bat (Myotis daubentonii) and has been implicated in two human cases. In Australia, the Australian bat lyssavirus has been detected in both megachiropteran and microchiropteran species, with two confirmed human infections reported so far^3^. However, these species may become more relevant as human occupation expands to areas where rabies viruses are endemic^4^.

Rabies exclusively affects mammals, causing acute and progressive encephalitis with a fatality rate of nearly 100%. Rabies transmission primarily occurs through bites or contact with the saliva of infected animals. Less frequently, transmission occurs through direct contact with infectious material on intact mucous membranes^5^. Globally, rabid dogs are estimated to be responsible for tens of thousands of deaths annually (approximately 55,000), particularly in Asia and Africa^6^ ^,^ ^7^.

The occurrence of rabies has been described for over four thousand years, and it was historically recognized as a disease that affects dogs and humans, “driving them mad.” The term “rabies” originates from the Latin word “rabere” (“rage” or “delirium”). In ancient Greece, rabies was called “lyssa” or “lytta,” meaning “madness.” Ancient civilizations hypothesized that dogs and wolves were influenced by demonic forces that altered their behavior. Others believed that the behavioral changes associated with rabies were caused by toxins present in the saliva of infected animals. Moreover, rabies was acknowledged as a significant concern in ancient legal frameworks, as evidenced by the Code of Eshnunna (23rd century BCE), which stipulated financial compensation in cases where a rabid animal caused human death^8^.

In 1881, Louis Pasteur successfully attenuated the virulence of the rabies virus by successively passing it through the central nervous systems of rabbits and subjecting their spinal cords to desiccation and treatment with potash. This procedure allowed for the development of a virus with reduced virulence and a consistent incubation period^9^. In 1884, Pasteur reported to the Paris Academy of Sciences that the virus exhibited decreased virulence after successive passages. The vaccine developed from this procedure was tested experimentally on animals. In 1885, Pasteur achieved a scientific breakthrough by administering the vaccine to a boy who had been bitten by a rabid dog, successfully preventing the onset of the disease. This landmark achievement represented the first effective treatment for rabies^10^. Human vaccines are produced from inactivated viruses cultivated in cell cultures. The vaccines are highly purified and safe and are recommended by the World Health Organization (WHO) for pre-exposure and post-exposure prophylaxis^11^. Pre-exposure prophylaxis with a rabies vaccine is recommended for individuals at continuous risk of rabies exposure, such as veterinarians, wildlife handlers, and professionals working in endemic areas. In Brazil, this applies to rural workers and ecotourism guides in high-risk areas. Post-exposure prophylaxis (PEP) is administered when an individual is bitten by an animal suspected to have rabies^12^ ^,^ ^13^. Rabies vaccines for animals are formulated using inactivated viruses and are used to induce immunity in dogs, cats, and livestock. These vaccines are typically used for prophylaxis and are administered according to veterinary calendars^14^. 

Epidemiological transmission of rabies is divided into four cycles: aerial (bat-associated), rural, urban (involving dogs and cats), and wildlife. These cycles may overlap depending on the presence of susceptible hosts^15^. Domestic dogs are the primary vectors of rabies transmission worldwide. In Brazil, common vampire bats (Desmodus rotundus) are currently considered the main wild reservoirs of the rabies virus, particularly in rural and sylvatic areas^16^. Other wildlife species, such as wild canids (Cerdocyon thous) and non-human primates (especially marmosets, Callithrix jacchus, and Callithrix penicillata), also play important roles in the transmission of the virus^17^ ^,^ ^18^.

Rabies is classified as a neglected disease and primarily affects vulnerable communities with an elevated risk of infection. Historically, most cases of human transmission from dogs have occurred in impoverished populations living and working under hazardous conditions. Although effective vaccines and human immunoglobulins are available for the prevention of rabies, these resources are inaccessible or financially prohibitive for most disadvantaged populations^19^. PEP remains the most effective intervention for preventing rabies in individuals exposed to the virus^12^ ^,^ ^13^. 

PEP protocols in Brazil are based on WHO guidelines but have been adapted to local populations owing to changes in disease epidemiology and shortages of rabies biologics. These changes were formalized in the updated national guidelines issued by the Ministry of Health in 2022, which emphasized rational use of the rabies vaccine, equine serum, and human rabies immunoglobulin, especially in light of the increasing incidence of rabies transmitted by wild animals^20^. Notably, PEP has been provided for free by Brazil’s Unified Health System since the establishment of the National Rabies Control Program in the 1970s^21^.

The Milwaukee Protocol, a treatment protocol for rabies developed in 2004, is globally disseminated and utilized, with adaptations based on geographical location. This protocol was first applied for the treatment of a vaccinated patient in the United States and has since undergone several modifications. The most recent version, version 6, was published in 2018 and was designed to facilitate rigorous control of electrolyte balance and management of vasoconstriction associated with rabies^22^. A notable complication of this treatment is hyponatremia, which may develop on the fifth day of hospitalization; however, prophylactic administration of fludrocortisone may mitigate this issue. The protocol suggests that one possible cause of electrolyte imbalance is dehydration, which can exacerbate brain injury. Therefore, regular monitoring of sodium level is crucial, and the recommended frequency of measurement is twice daily^23^. According to the Medical College of Wisconsin, antiviral treatment should ideally be administered in the early stage of the disease. The Milwaukee Protocol recommends initiating antiviral therapy within five days, even in the absence of a definitive rabies diagnosis. 

In 2008, the Recife Protocol, an adaptation of the Milwaukee Protocol, was implemented in Brazil due to the unavailability of the drugs recommended in the original protocol. This protocol was applied for the treatment a 15-year-old boy who was bitten by a bat and sought medical care three weeks after the incident. After 35 days of hospitalization, the patient was cured. In 2018, a second patient in Brazil survived rabies after receiving treatment administered using the Recife Protocol; however, the patient died seven years later in 2025^24^. Both protocols are focused on deep sedation, administration of antiviral drugs, continuous monitoring of electrolyte balance, and management of vasoconstriction associated with the disease. The Recife Protocol specifically highlights the risks of both hypernatremia and hyponatremia, which require careful monitoring and intervention^25^.

The primary factor that contributes to the survival of patients with rabies is the level of intensive care provided. However, increasing public awareness of rabies treatment is crucial because rabies is often perceived as an inevitably fatal disease; consequently, affected persons rarely seek lifesaving treatment. Over the years, management of rabies typically only involved administration of palliative treatments aimed at alleviating the symptoms and conditions of patients^26^.

This study was conducted to examine the epidemiological patterns of human rabies in Brazil between 2001 and 2025, with particular focus on transmission sources, timing and use of PEP, and clinical outcomes. This analysis addresses the transition from urban to wildlife-associated transmission, the impact of animal vaccination campaigns on canine rabies control, and the circulation of distinct rabies virus variants. Additionally, it highlights rare cases of patient survival and the ongoing challenges related to rabies prevention and clinical management. The findings of this study underscore the importance of ensuring equitable access to vaccines, immunoglobulins, and timely medical care, as well as the need for sustained surveillance and public health interventions to reduce the lethality of this preventable disease.

## METHODS

The data used for this study were obtained from the Brazilian Ministry of Health via the DATASUS platform, which consolidates information on mandatory notifications and epidemiological investigations of diseases and health-related events (http://tabnet.datasus.gov.br/cgi/deftohtm.exe?sinannet/cnv/raivabr.def). Systematic use of this database enabled characterization of the spatial and temporal distribution of rabies cases across the country, facilitating the identification of epidemiological patterns and assessment of risks in different populations. The database also provides data on PEP administration through the TabWin online tool (https://datasus.saude.gov.br/transferencia-de-arquivos/). 

The variables analyzed in this study included the cases of infections at regional and national levels, species responsible for transmission, and year of notification. Data on vaccination of dogs and cats were obtained from the National Immunization Program Information System and the Secretariat of Health and Environmental Surveillance, Ministry of Health. Official websites and technical notes from the Brazilian Ministry of Health were also accessed to obtain additional data, particularly data for recent years (https://www.gov.br/saude/pt-br/assuntos/saude-de-a-a-z/r/raiva).

In addition to the epidemiological data analysis, a comprehensive literature review was conducted using public scientific databases, which included PubMed, SciELO, and Google Scholar. The review was focused on studies on rabies epidemiology, transmission cycles, environmental impacts, and control policies in Brazil and worldwide. Articles published between 2001 and 2025, particularly those that provided insights into the evolution of transmission patterns, therapeutic approaches, and prevention strategies, were included in the review.

## RESULTS

**FIGURE 1:**
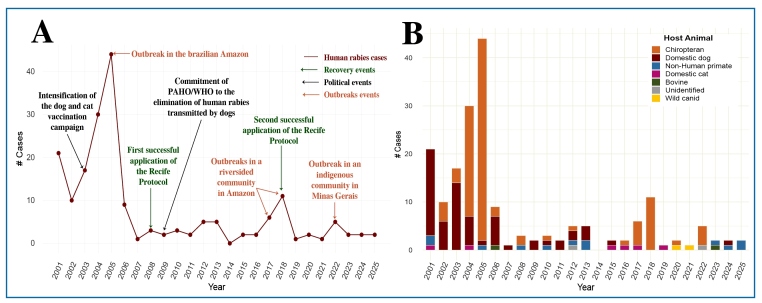
(A): Evolution of the cases of human rabies in Brazil between 2001 and 2025, highlighting significant events. Cases of patient recovery are indicated in green, political events related to rabies control are indicated in black, and outbreaks recorded during the analyzed period are indicated in red. The data reflect the relationship between control measures, international commitments, and the persistence of outbreaks in certain regions. (B): Distribution of rabies cases according to year and host animal between 2001 and 2025. The rabies cases are grouped according to host animals into the following categories: cases transmitted by bats (Chiropteran), which exhibited increasing frequency over time, are depicted in light orange; cases transmitted by domestic dogs are shown in dark red; cases transmitted by domestic cats, which highlight a reduction in cases transmitted by domestic animals, are indicated in purple; cases transmitted by non-human primates are shown in blue; cases transmitted by wild canids (fox), which indicate a shift in the transmission profile towards wild animals, are shown in yellow; cases transmitted by bovines, which showed reduced frequency over time, reflecting the success of the National Rabies Control Program for Herbivores, are indicated in dark green; and unidentified cases (ignored/not identified), possibly due to incomplete or imprecisely categorized data, are indicated in gray.

A total of 188 human rabies cases were reported in Brazil between 2001 and 2025 (7.52 cases per year). Of these, 36.17% (68/188) occurred in the North, 50.53% (95/188) in the Northeast, 3.19% (6/188) in the Central-West, 9.57% (18/188) in the Southeast, and 0.53% (1/188) in the South. No cases were reported in the states of Amapá, Rio Grande do Sul, and Paraná during the study period. Figure 1A shows the species responsible for the transmission of human rabies each year. Dogs were the most common source of transmission (34.04%, 64/188), followed by cats (3.19%, 6/188), bats (53.19%, 100/188), non-human primates (6.38%, 12/188), other animals (2.13%, 4/188), and unknown sources (1.06%, 2/188).

Evaluation of the data from the SINAN-NET platform (Table 1) showed that 163 cases had information on sex, age group, and/or type of exposure. The discrepancy (n = 25) between the 188 reported cases and the 163 documented in the database is because 25 cases were not included in Brazil’s Notifiable Diseases Information System (SINAN). The SINAN database only contains data on confirmed human rabies cases recorded up to 2021. However, our study includes data collated up to 2025. These 25 additional cases were later reported by the Ministry of Health and disclosed exclusively through the Ministry’s official webpage on human rabies but were not updated in the SINAN system. Consequently, the patients’ demographic data are not accessible through standard epidemiological databases.

**TABLE 1:** Distribution of cases according to sex, age group, and type of exposure (2001-2021). The data presented were obtained through the SINAN-NET platform. Some data are missing due to information gaps in the database, which has not been updated since 2021.

Variable	Category	Total
Sex	Male	107
	Female	56
Age group	< 1	0
	1-4	24
	5-9	27
	10-14	34
	15-19	12
	20-39	42
	40-59	18
	60-64	1
	65-69	1
	70-79	1
	80+	1
	Blank	0
Type of exposure	Inoculation	56
	No inoculation	59
	Unknown/Not reported	48

Of the 163 patients, 103 were males and 56 were females, indicating that men were disproportionately affected by rabies during the study period. The age group distribution of the patients is as follows: 34 patients in the 10-14 years group, 42 in the 20-39 years group, 27 in the 5-9 years group, and 24 in the 1-4 years group. These findings indicate that children and young adults, especially those aged 10-14 years, were most commonly affected. Notably, the older age groups (60-64, 65-69, 70-79, and 80+) included only one patient each. No case of an infant under the age of one year was reported during the study period. Regarding types of exposure, inoculation exposure (e.g., bites/scratches) was reported in 56 cases, whereas no inoculation (likely indirect contact or other routes) occurred in 59 cases. In addition, 48 patients had unknown or unreported exposure, highlighting a significant limitation of the dataset.

Human rabies transmission peaked in 2004 and 2005, with 75 cases reported within this period (Figure 1A). Of these, 39 occurred in Pará and 28 in Maranhão states. These cases were primarily attributed to outbreaks caused by vampire bats (Desmodus rotundus). Additionally, outbreaks were recorded in 2018 and 2022 in riverside communities of the Brazilian Amazon and among indigenous populations in Minas Gerais, respectively.

The incidence of human rabies significantly declined over the years and the transmission dynamics changed from domestic animals to bats (Figures 1A and 1B). This change is attributed to the success of the National Rabies Prophylaxis Program for Domestic Animals, which was established in 1973 to enable the implementation of rabies vaccination campaigns for dogs and cats across Brazil. The program was intensified in 2003 and aligned with the goals of international bodies, such as the PAHO/WHO, to eliminate dog-transmitted rabies in the Americas by 2030. However, recent outbreaks that involved wildlife transmission, such as that recorded in an indigenous community in Minas Gerais in 2022 (Figure 1B), highlight the need for continuous surveillance, particularly in vulnerable populations. 

Table 2 presents the available data on the antigenic or genetic variants of the rabies viruses identified in human rabies cases reported in Brazil from 2001 to 2025. Unfortunately, reports of rabies virus variants only became available from 2010 onwards. Therefore, the retrospective interpretation of the cases reported before 2010 was limited. Nevertheless, the available data indicate a predominance of the AgV2 variant in dog-associated transmissions and AgV3 in cases linked to bats and secondary hosts such as cats. These patterns emphasize the role of the domestic environment as a key interface in the rabies transmission chain, particularly when wildlife-maintained variants (e.g., those circulating in bats) are transmitted to domestic animals, such as cats, which in turn serve as sources of human infection.

**TABLE 2:** Human rabies cases in Brazil categorized according to year, state, municipality, host animal, and virus variant (2010-2025). Consistent data regarding the host animal and the antigenic variant, as provided by the Brazilian Ministry of Health, are available only from 2010 onwards.

Year	UF	Municipality	Host animal	Antigenic variants
2001	SP	Dracena	Domestic cat	AgV3
2010	CE	Ipu	Non-human primate	AgV Marmoset
2010	CE	Chaval	Domestic dog	AgV2
2010	RN	Frutuoso Gomes	Chiropteran	AgV3
2011	MA	Paço do Lumiar	Domestic dog	AgV2
2011	MA	São José do Ribamar	Domestic dog	AgV2
2012	CE	Jati	Non-human primate	AgV Marmoset
2012	MA	São Luis	Domestic dog	AgV2
2012	MA	São Luis	Domestic dog	AgV2
2012	MG	Rio Casca	Chiropteran	AgV3
2012	MT	Tapurah	Unidentified	Unknown
2013	PI	Pio IX	Non-human primate	AgV Marmoset
2013	MA	São José do Ribamar	Non-human primate	AgV Marmoset
2013	MA	Humberto de Campos	Domestic dog	AgV2
2013	MA	Mirinzal	Domestic dog	AgV2
2013	PI	Parnaíba	Domestic dog	AgV2
2015	MS	Corumbá	Domestic dog	AgV1
2015	PB	Jacaraú	Domestic cat	AgV3
2016	RR	Boa Vista	Domestic cat	AgV3
2016	CE	Iracema	Chiropteran	AgV3
2017	PE	Recife	Domestic cat	AgV3
2017	AM	Barcelos	Chiropteran	AgV3
2017	AM	Barcelos	Chiropteran	AgV3
2017	AM	Barcelos	Chiropteran	AgV3
2017	BA	Paramirim	Chiropteran	AgV3
2017	TO	Ponte Alta do Tocantins	Chiropteran	AgV3
2018	PA	Melgaço	Chiropteran	AgV3
2018	PA	Melgaço	Chiropteran	AgV3
2018	PA	Melgaço	Chiropteran	AgV3
2018	PA	Melgaço	Chiropteran	AgV3
2018	PA	Melgaço	Chiropteran	AgV3
2018	PA	Melgaço	Chiropteran	AgV3
2018	PA	Melgaço	Chiropteran	AgV3
2018	PA	Melgaço	Chiropteran	AgV3
2018	PA	Melgaço	Chiropteran	AgV3
2018	PA	Melgaço	Chiropteran	AgV3
2018	SP	Ubatuba	Chiropteran	AgV3
2019	SC	Gravatal	Domestic Cat	AgV3
2020	PB	Riacho dos Cavalos	Wild canid (fox)	AgV Wild canid
2020	RJ	Angra dos reis	Chiropteran	AgV3
2021	MA	Chapadinha	Wild canid (fox)	AgV Wild canid
2022	DF	Brasilia	Unidentified	AgV3
2022	MG	Bertópolis	Chiropteran	AgV3
2022	MG	Bertópolis	Chiropteran	AgV3
2022	MG	Bertópolis	Chiropteran	AgV3
2022	MG	Bertópolis	Chiropteran	AgV3
2023	CE	Cariús	Non-human primate	AgV Marmoset
2023	MG	Mantena	Bovine	AgV3
2024	PI	Piripiri	Non-human primate	AgV Marmoset
2024	TO	Alvorada	Domestic dog	AgV3
2025	CE	Jucás	Non-human primate	AgV Marmoset
2025	PE	Santa Maria do Cambucá	Non-human primate	AgV Marmoset

**FIGURE 2:**
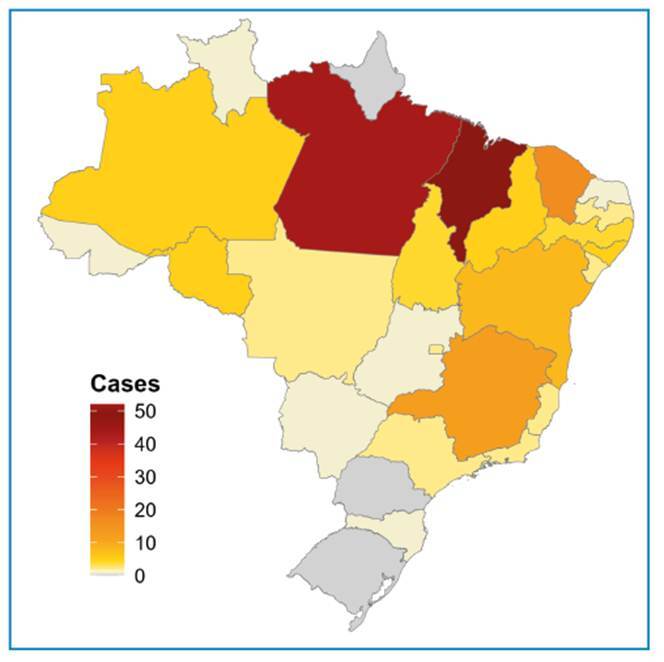
Spatial distribution of human rabies cases in Brazil according to state. The map displays the total number of registered cases, with a color gradient ranging from yellow (indicating a lower number of cases) to dark red (indicating a higher number of cases). States shown in grey have no available records for the analyzed period.

The spatial distribution of human rabies cases across the country is illustrated in Figure 2. The highest incidence was observed in the northern region, particularly in the state of Pará, which experienced multiple significant outbreaks. In contrast, the south and southeast regions reported comparatively low numbers of cases. This regional variation may reflect differences in surveillance capacity, animal reservoir dynamics, and implementation of prevention strategies, including vaccination campaigns.

Efforts to control canine rabies through mass vaccination have played a central role in reducing dog-transmitted rabies. Data retrieved from the National Immunization Program Information System (Supplementary Table S1) cover the period from 2012 to 2017 and is focused solely on dogs. Data on cat vaccination coverage remains unavailable. The states of Rio Grande do Sul and Santa Catarina discontinued their canine rabies vaccination campaigns in 1995, whereas the state of Paraná limited its vaccination efforts to the municipalities bordering Paraguay until 2015. The most recent nationwide vaccination coverage reported in 2024 is 60.4%, indicating potential weakening of the rabies prevention infrastructure.

PEP data for the period between 2007 and 2025 highlight critical gaps in the clinical response to human rabies. Structured PEP data are available in DATASUS for 2007-2021. However, the data for 2022-2025 is limited to individual case reports that lack consistent documentation of PEP administration. Of the 57 confirmed cases reported during this period, PEP was administered in only 17 cases; PEP was not administered in 27 cases, and data on PEP administration was not included in 12 cases (Table 3). The average interval between exposure and symptom onset was 38.6 days (median: 32 days), and the mean number of days from exposure to PEP administration was 30.6 days (median: 30.5 days). Seven patients received anti-rabies vaccination only after symptom onset, which is in contrast to the current clinical recommendations. Notably, the mean number of days from symptom onset to death was 17, a finding that highlights the rapid progression of this neglected disease. There was no detailed information on the number of times or specific cases in which the Recife Protocol was applied. However, two cases with favorable outcomes following treatment were reported: one in 2008 in Pernambuco and one that involved a 14-year-old male, which was the first documented application of the Recife Protocol. In this case, symptom onset occurred 27 days after exposure, and PEP was initiated within four days. The second survivor was a 15-year-old male from Amazonas who was diagnosed in 2017. The patient presented with symptoms at 98 days post-exposure, and PEP was administered nearly three months post-exposure. Although the patient received treatment under the Recife Protocol and initially survived, he subsequently died of disease-related neurological sequelae (Table 3). 

**TABLE 3:** Information regarding the treatment administered in each case of human rabies (2007-2021). Consistent data have been available on the SINAN-NET platform since 2007. Reliable records collated before 2007 are lacking, and the platform has not been updated since 2021.

Year	State	Age	Sex	Prophylaxis	RIG infiltration at the wound site	Exposure to symptoms*	Exposure to PEP*	Duration from symptom onset to death*
2007	MA	19	M	NR	NR	NR	NR	10
2008**	PE	15	M	ARV	NR	27	4	
2008	CE	16	F	ARV; RIG	Unrealized	53	53	NR
2008	DF	12	M	Untreated	NR	31	Untreated	30
2009	MA	26	M	ARV; RIG	Fully realized	3	4	NR
2009	PI	12	M	ARV; RIG	Partial	38	41	4
2010	RN	49	M	Untreated	NR	54	Untreated	8
2010	CE	11	M	Untreated	NR	60	Untreated	18
2010	CE	26	M	Untreated	NR	77	Untreated	12
2011	MA	39	M	ARV; RIG	Unrealized	0	6	4
2011	MA	58	M	RIG	Unrealized	6	NR	12
2012	MA	35	F	RIG	Partial	28	34	18
2012	CE	9	M	Untreated	NR	20	Untreated	16
2012	MG	32	M	Untreated	NR	54	Untreated	18
2012	MA	33	M	Untreated	NR	61	Untreated	11
2012	MT	21	F	Untreated	NR	69	Untreated	16
2013	PI	31	M	ARV; RIG	Unrealized	3	3	3
2013	MA	4	M	ARV; RIG	NR	30	30	27
2013	MA	33	M	RIG	NR	NR	NR	21
2013	MA	25	M	Untreated	NR	60	Untreated	24
2013	PI	50	M	Untreated	NR	75	Untreated	2
2015	MS	39	M	ARV	NR	59	62	33
2015	PB	1	M	NR	NR	NR	NR	NA
2016	CE	37	M	NR	NR	NR	NR	15
2016	RR	14	M	Untreated	NR	30	Untreated	NA
2017	BA	46	M	ARV; RIG	NR	24	31	9
2017**	AM	14	M	ARV; RIG	Unrealized	98	90	
2017	TO	5	M	Untreated	NR	0	Untreated	11
2017	PE	35	F	Untreated	NR	23	Untreated	7
2017	AM	10	F	Untreated	NR	52	Untreated	24
2017	AM	17	M	Untreated	NR	100	Untreated	NR
2018	SP	24	M	ARV; RIG	Unrealized	9	12	56
2018	PA	4	F	Untreated	NR	10	Untreated	17
2018	PA	9	M	Untreated	NR	14	Untreated	5
2018	PA	2	M	Untreated	NR	15	Untreated	4
2018	PA	9	M	Untreated	NR	46	Untreated	15
2018	PA	39	M	Untreated	NR	54	Untreated	15
2018	PA	8	F	Untreated	NR	64	Untreated	24
2018	PA	3	M	Untreated	NR	NR	Untreated	14
2018	PA	11	F	Untreated	NR	NR	Untreated	17
2018	PA	10	F	Untreated	NR	NR	Untreated	4
2018	PA	8	M	Untreated	NR	NR	Untreated	36
2019	SC	58	F	ARV; RIG	NR	0	59	NA
2020	RJ	14	M	RIG	Partial	26	0	37
2020	PB	67	F	Untreated	NR	62	Untreated	34
2021	MA	2	M	Untreated	NR	33	Untreated	47

NR: Not reported; Untreated: Did not receive anti-rabies treatment; ARV: anti-rabies vaccination; RIG: rabies immunoglobulin. *Number of days; **Favorable outcome.

Due to the lack of standardized and complete clinical records before 2007, cases reported earlier were excluded from the analysis of clinical management and prophylactic response.

## DISCUSSION

Transmission of rabies by wild animals remains a significant public health concern, particularly because of the increasing rate of human-wildlife contact. The increased contact between humans and bats is partly driven by human activities such as logging. In 2004, the municipality of Portel reported the environmental effects of logging activities, which changed ecosystems and favored the migration of bats to areas inhabited by humans. Ecological models indicate that climate change can modify the spatial distribution and population dynamics of wild animals, directly influencing the incidence of human rabies. This trend was observed in the outbreaks documented in 2004, 2017, and 2022^27^. 

Bats, particularly Desmodus rotundus, commonly migrate to human-occupied areas because of habitat and food shortages caused by environmental changes such as deforestation and wildfires^28^. Four cases of cat-transmitted human rabies reported between 2015 and 2019 were caused by the AgV3 variant, which is associated with bats. This phenomenon, known as secondary rabies transmission, occurs when cats get infected through contact with bats and subsequently transmit the disease to humans. Such cases emphasize the need for continuous monitoring of wildlife populations. 

Studies conducted in Brazil have demonstrated the presence of AgV3 in frugivorous bats of the Artibeus lituratus species in the states of Tocantins, Rondônia, and Roraima, thereby providing evidence of the ecological adaptability of the AgV3 variant. These findings indicate that AgV3 is not restricted to hematophagous bats, but may also circulate among non-hematophagous species, possibly due to cohabitation of roosts and ecological interactions among different bat species^29^.

As of the time this article was written (2025), two cases of human rabies had been reported in Brazil (in the states of Ceará and Pernambuco), both transmitted by marmosets (Callithrix spp). These two cases raise concerns regarding human contact with non-human primates, which were not previously been considered significant rabies reservoirs in Brazil. In addition, this development reinforces the need for increased surveillance for identification of infected animals in urban areas and evaluation of the roles of wild animals in viral transmission^30^.

Cases of rabies transmission by non-human primates are common in the northeastern region of Brazil. Illegal ownership of marmosets as pets is widespread in this region, and bite-related incidents have often been reported^31^. Marmosets were responsible for ten cases of human rabies reported in Brazile between 2001 and 2025, all recorded in the northeast region. This highlights the risks posed by close interactions between marmosets and humans.

Frequent contact between humans and marmosets facilitates viral transmission. Aguiar et al. (2011) identified a rabies virus variant exclusively associated with marmosets, with no antigenic or genetic relationship to rabies strains found in bats or terrestrial mammals in the Americas. This finding highlights a possible independent rabies transmission cycle among primates in Brazil. However, further investigation is required to confirm this hypothesis^32^.

Analysis of the available data on rabies vaccination for domestic animals revealed a persistent lack of information on vaccination coverage in cats. This gap may be attributed to limited public awareness or misconceptions regarding the role of felines in the transmission of rabies.

Between 2001 and 2013, 21 cases of rabies transmitted by domestic dogs were reported in Maranhão, Brazil. A 2014 study on knowledge of rabies among health professionals and community members in five municipalities of Maranhão revealed that 39.3% of the professionals interviewed had not received training on suspected rabies cases, and that 22% were unaware of the virus^33^. Between 2000 and 2009, national rabies vaccination campaigns in Brazil resulted in an average canine vaccination coverage of 86% (range: 81%-94%). Approximately 21.4 million animals were vaccinated annually, 82% of which were dogs. In addition to national efforts, targeted intensification campaigns were conducted in high-risk areas, and a mean coverage of 75% (range: 70%-81%) was achieved. Currently, most cases of human rabies in Brazil are associated with wildlife reservoirs, especially bats and non-human primates. This highlights the need for extensive surveillance and prevention strategies beyond the domestic transmission cycle^34^.

PEP is a key strategy for reducing the incidence of rabies-related deaths. PEP includes vaccination and, depending on the location of the bite and severity of exposure to the virus, administration of rabies immunoglobulin (RIG). The Essen regimen is the gold standard for PEP, and it comprises the administration of intramuscular immunoglobulin at the wound site, along with five doses of the rabies vaccine on days 0, 3, 7, and 14. Although PEP has high success rates, discontinuing the regimen prematurely can result in insufficient protection against the virus. Unfortunately, PEP stockouts frequently occur in many endemic regions because of the high cost of the treatment, limited awareness and procurement, and the high incidence of bite-related cases that require PEP^35^. 

In some cases analyzed in the present study, the interval between symptom onset and death was as short as three days, suggesting critical delays in seeking medical care. These delays may be attributed to low risk perception, the nonspecific nature of early rabies symptoms, or limited awareness of wildlife as a potential source of infection. In seven cases, anti-rabies vaccination was initiated after the onset of clinical symptoms, indicating deviations from established clinical protocols and potentially reflecting knowledge gaps among healthcare providers or patients. Additionally, structural barriers to healthcare access in remote or underserved areas may have contributed to the delayed administration or complete omission of PEP.

The consistent lack of treatment-related data in national databases hinders assessment of the clinical management of human rabies and underscores an urgent need to enhance the quality and completeness of epidemiological reporting within surveillance systems. 

Although rare, breakthrough infections can occur even when PEP is administered correctly, particularly in immunosuppressed individuals. However, results from previous studies indicate that most failures are associated with delays, improper administration of rabies immunoglobulin, or health system gaps, rather than biological or physiological limitations^36^. These findings highlight the importance of timely data collection and targeted education to inform public health policies, improve risk communication strategies, and guide resource allocation in underserved regions.

Technological advances have enabled the development of artificial intelligence (AI)-based tools for combating the spread of infectious diseases worldwide. These technologies are now being implemented in Brazil^37^ ^,^ ^38^. In addition, AI has been used for predictive analysis of rabies and to guide decision-making for infection control in Morocco and Haiti, yielding highly accurate results, particularly in regions with limited surveillance resources^39^ ^,^ ^40^. However, no AI-based research on rabies control in Brazil has been conducted to date. Adoption of AI by research groups in Brazil could be beneficial for improved surveillance of rabies cases in the country. 

Currently, research groups are exploring preventive strategies against the rabies virus ^41^ ^-^ ^43^. However, although previous studies have yielded promising results, research on implementation of these preventive strategies in Brazil remains limited^44^ ^-^ ^46^. Over the past decade, in silico assays have played a crucial role in advancing the development of drugs such as Darunavir and Compound 13b for the treatment of HIV and SARS-CoV-2 infections, respectively^47^ ^,^ ^48^. The increasing availability of in silico tools and computational resources, which were once considered impractical, has significantly accelerated drug development and reduced the need for substantial financial investment in therapies^49^ ^,^ ^50^. Therefore, applying these methodologies to the development of rabies antivirals in Brazil is a promising approach that should be explored in future research.

## CONCLUSION

The findings of this study indicate a decline in human rabies cases and a shift in the transmission profile of the disease over the past 20 years, owing to the success of the National Rabies Prophylaxis Program for Domestic Animals. However, the existing vaccination programs should be extended beyond dogs and cats to include livestock and wild animals in high-risk areas. In addition, public awareness campaigns must be enhanced to educate communities on the risks associated with handling wild animals and the importance of PEP. Furthermore, development of more accessible and effective treatment strategies is vital for ensuring better access to rabies immunoglobulins and antiviral therapies.

Strengthening rabies surveillance systems is crucial, particularly in regions with high rates of wildlife transmission. The commitment of international organizations, such as the PAHO/WHO, to the elimination of human deaths from canine rabies by 2030 represents a significant milestone in ongoing rabies control efforts. However, addressing the emerging challenges of wildlife transmission requires continuous research, epidemiological surveillance, and adaptation of public health policies to mitigate the risk of future outbreaks.

## References

[1] Banyard AC, Fooks AR (2020). Rabies Life Cycle, Transmission and Pathogenesis. In: Rabies and Rabies Vaccines.

[2] Walker PJ, Freitas-Astúa J, Bejerman N, Blasdell KR, Breyta R, Dietzgen RG (2022). ICTV Virus Taxonomy Profile: 2022. J Gen Virol.

[3] (2011). Advances in Virus Research.

[4] Hayman DTS, Fooks AR, Marston DA, Garcia-R JC (2016). The Global Phylogeography of Lyssaviruses - Challenging the “Out of Africa” Hypothesis. PLoS Negl Trop Dis.

[5] Sreenivasan N, Li A, Shiferaw M, Tran CH, Wallace R, Blanton J (2019). Overview of rabies post-exposure prophylaxis access, procurement and distribution in selected countries in Asia and Africa, 2017-2018. Vaccine.

[6] Putra AAG, Hampson K, Girardi J, Hiby E, Knobel D, Mardiana IW (2013). Response to a rabies epidemic, Bali, Indonesia, 2008-2011. Emerg Infect Dis.

[7] WHO (2005). WHO Expert Consultation on Rabies: First Report.

[8] Kotait I, Carrieri ML, Takaoka NY (2009). Manual de Procedimentos para Diagnóstico da Raiva.

[9] Flint SJ, Enquist LW, Krug RM, Racaniello VR, Skalka AM (2000). Principles of Virology: Molecular Biology, Pathogenesis, and Control.

[10] Vallery-Radot R (1911). The Life of Pasteur.

[11] São Paulo SM de S (2025). Vacina antirrábica humana (VAR).

[12] (2018). WHO Expert Consultation on Rabies: Third Report.

[13] Schneider MC, Min KD, Romijn PC, De Morais NB, Montebello L, Manrique Rocha S (2023). Fifty Years of the National Rabies Control Program in Brazil under the One Health Perspective. Pathogens.

[14] Vacina Antirrábica (2022). Ministério da Agricultura e Pecuária.

[15] Singh R, Singh KP, Cherian S, Saminathan M, Kapoor S, Manjunatha Reddy GB (2017). Rabies - epidemiology, pathogenesis, public health concerns and advances in diagnosis and control: a comprehensive review. Veterinary Quarterly.

[16] Vargas A, Romano APM, Merchán-Hamann E (2019). Human rabies in Brazil: a descriptive study, 2000-2017. Epidemiol Serv Saude.

[17] Benavides JA, Raghavan RK, Boere V, Rocha S, Wada MY, Vargas A (2022). Spatio-temporal dynamics of rabies and habitat suitability of the common marmoset Callithrix jacchus in Brazil. PLoS Negl Trop Dis.

[18] Sacramento DRS, Albas A, Durigon GS, Montebello L, Albas KH (2016). Rabies virus related to vampire bats (Desmodus rotundus) isolated from a crab-eating fox (Cerdocyon thous) in Southeast Brazil. JSM Trop Med Res.

[19] Swedberg C, Bote K, Gamble L, Fénelon N, King A, Wallace RM (2024). Eliminating invisible deaths: the woeful state of global rabies data and its impact on progress towards 2030 sustainable development goals for neglected tropical diseases. Front Trop Dis.

[20] Brasil. Ministério da Saúde (2022). Nota Técnica nº 8/2022-CGZV/DEIDT/SVS/MS.

[21] Castro MC, Massuda A, Almeida G, Menezes-Filho NA, Andrade MV, de Souza Noronha KVM (2019). Brazil’s unified health system: the first 30 years and prospects for the future. Lancet.

[22] Medical College of Wisconsin (2018). Milwaukee Protocol.

[23] Ledesma LA, Lemos ERS, Horta MA (2020). Comparing clinical protocols for the treatment of human rabies: the Milwaukee protocol and the Brazilian protocol (Recife). Rev Soc Bras Med Trop.

[24] DST Júnior, Marques MSV, de Oliveira RC (2024). Adapted Milwaukee protocol for rabies treatment in a Brazilian indigenous child: case report. Virol J.

[25] Nadeem M, Panda PK (2020). Survival in human rabies but left against medical advice and death followed - Community education is the need of the hour. J Family Med Prim Care.

[26] Brasil. Ministério da Saúde (2016). Vigilância em saúde de zoonoses: desafios e perspectivas.

[27] Neves JMM, Belo VS, Catita CMS, Oliveira BFA de, Horta MAP (2024). Modeling of Human Rabies Cases in Brazil in Different Future Global Warming Scenarios. Int J Environ Res Public Health.

[28] Torres-Mejía X, Pérez-Rivero JJ, Olvera-Vargas LA, Barragán-Hernández EÁ, Martínez-Maya JJ, Aguilar-Setién Á (2021). La coexistencia de Desmodus rotundus con la población humana en San Luis Potosí, México. Rev Mex Cienc Pecu.

[29] Cunha TCA da S da, Silva FS da, Silva SP da, Ribeiro Cruz AC, Paiva FA dos S, Casseb LMN (2023). Phylogenetic analysis of rabies surveillance samples from north and northeast Brazil. Front Vet Sci.

[30] Batista Moutinho FF, Mendonça da Silva Correa D, Marcanth N, Moura Azevedo Nunes V, Villas Boas Borges F (2020). Vigilância de epizootias em primatas não humanos em Niterói, RJ, BRASIL. Rev Bras Geogr Médica Saúde.

[31] Favoretto SR, de Mattos CC, de Mattos CA, Campos ACA, Sacramento DRV, Durigon EL (2013). The emergence of wildlife species as a source of human rabies infection in Brazil. Epidemiol Infect.

[32] Aguiar TD de F, Costa EC, Rolim BN, Romijn PC, Morais NB de, Teixeira MF da S (2011). Risks of transmitting rabies virus from captive domiciliary common marmoset (Callithrix jacchus) to human beings, in the metropolitan region of Fortaleza, state of Ceará, Brazil. Rev Soc Bras Med Trop.

[33] Saraiva DS, Thomaz EBAF, Caldas A de JM (2014). Raiva humana transmitida por cães no Maranhão: avaliação das diretrizes básicas de eliminação da doença. Cad Saúde Colet.

[34] Wada MY, Rocha SM, Maia-Elkhoury ANS (2011). Situação da Raiva no Brasil, 2000 a 2009. Epidemiol Serv Saude.

[35] Wambura G, Mwatondo A, Muturi M, Nasimiyu C, Wentworth D, Hampson K (2019). Rabies vaccine and immunoglobulin supply and logistics: Challenges and opportunities for rabies elimination in Kenya. Vaccine.

[36] Whitehouse ER, Mandra A, Bonwitt J, Beasley EA, Taliano J, Rao AK (2023). Human rabies despite post-exposure prophylaxis: a systematic review of fatal breakthrough infections after zoonotic exposures. Lancet Infect Dis.

[37] Valter R, Oliveira M, Vitorino W, Neuman J, Albuquerque S, Andrade LOM (2021). Intelligent epidemiological surveillance in the Brazilian semiarid.

[38] Silva JFA, Izbicki R, Bastos LS, Soares GP (2024). Monitoring viral infections in severe acute respiratory syndrome patients in Brazil. In: Contributions to Statistics.

[39] Ahamjik I, Agbani A, Abik M, Khayli M, Galzim N, Berrada J (2024). Contribution of artificial intelligence for understanding animal rabies epidemiology in Morocco: What are the perspectives of an innovative and predictive approaches?. One Health.

[40] Keshavamurthy R, Boutelle C, Nakazawa Y, Joseph H, Joseph DW, Dilius P (2024). Machine learning to improve the understanding of rabies epidemiology in low surveillance settings. Sci Rep.

[41] Rahmati S, Zandi F, Ahmadi K, Adeli A, Rastegarpanah N, Amanlou M (2025). Computational structure-based design of antiviral peptides as potential protein-protein interaction inhibitors of rabies virus phosphoprotein and human LC8. Heliyon.

[42] Zhan J, Chakraborty S, Sethi A, Mok YF, Yan F, Moseley GW, Gooley PR (2025). Analysis of mechanisms of the rabies virus P protein-nucleocapsid interaction using engineered N-protein peptides and potential applications in antivirals design. Antiviral Res.

[43] De Pijper CA, van Thiel PPAM, van de Beek D, Brouwer MC, Aronica E, Juffermans NP (2025). Rabies in humans: A treatment approach. Travel Med Infect Dis.

[44] Ono EAD, Iamamoto K, Castilho JG, Carnieli P, Oliveira R de N, Achkar SM (2013). In vitro and in vivo inhibition of rabies virus replication by RNA interference. Braz J Microbiol.

[45] Agostinho WC, Brandão PE (2020). Intracerebral transfection of anti-rabies virus antibodies is an effective therapy for rabies. J Neurovirol.

[46] de Melo GD, Sonthonnax F, Lepousez G, Jouvion G, Minola A, Zatta F (2020). A combination of two human monoclonal antibodies cures symptomatic rabies. EMBO Mol Med.

[47] Chen CC, Yu X, Kuo CJ, Min J, Chen S, Ma L (2021). Overview of antiviral drug candidates targeting coronaviral 3C-like main proteases. FEBS J.

[48] Mahto MK, Yellapu NK, Kilaru RB, Chamarthi NR, Bhaskar M (2014). Molecular designing and in silico evaluation of darunavir derivatives as anticancer agents.. Bioinformation.

[49] Dokhale S, Garse S, Devarajan S, Thakur V, Kolhapure S (2024). Computational Methods for Rational Drug Design.

[50] de Sousa LLF, Guilardi MD, Martins JO, Alves BSS, Tibo LHS, da Silva-Antunes P (2025). Phylogenetic inferences reveal multiple intra- and interhost genetic diversity among bat rabies viruses circulating in northeastern Brazil. One Health Outlook.

