# Supplementary material for: Two Decades of Human Rabies in Brazil: Epidemiological Trends, Emerging Risks and Treatment Challenges
Source: Rev Soc Bras Med Trop. 2025 Aug 1;58:e0110-2025. doi: 10.1590/0037-8682-0110-2025 (PMC12316195; doi:10.1590/0037-8682-0110-2025)
Supplement: Supplementary file 1 [file 1678-9849-rsbmt-58-e0110-2025-supp1.pdf]

SUPPLEMENTARY TABLE S1: Vaccination Coverage and Doses Administered Against Rabies in Dogs and Cats, Brazil (2012–2017).

UF	2012		2013		2014		2015		2016		2017	
	Dogs	Cats	Dogs	Cats	Dogs	Cats	Dogs	Cats	Dogs	Cats	Dogs	Cats
	Doses (VC(%))	Doses	Doses (VC(%))	Doses	Doses (VC(%))	Doses	Doses (VC(%))	Doses	Doses (VC(%))	Doses	Doses (VC(%))	Doses
AC	71316 (63.78)	45731	48524 (43.39)	14.31	42737 (38.22)	9821	101776 (89.18)	27329	86876 (75.19)	19.244	104691 (90.61)	22.236
AM	327667 (80.11)	105305	361.24 (88.31)	108447	372.32 (89.43)	122.432	383436 (88.86)	124936	377135 (86.19)	130.167	387012 (87.53)	135.914
AP	0 (0)	0	8438 (10.47)	2.646	41002 (60.64)	11.89	38179 (55.75)	11832	48042 (52.22)	14.522	60082 (65.3)	15.957
PA	536.59 (48.95)	145561	1001111 (87.87)	301.111	986435 (90.85)	287.04	948594 (83.2)	257602	933234 (5.25)	267.557	1046370 (90.82)	279.792
RO	101867 (25.46)	22307	201642 (70.94)	50.178	256107 (79.76)	55.209	314.98 (95.18)	68026	298784 (92.98)	66927	331724 (100.49)	72.19
RR	0 (0)	0	6943 (12.25)	1.015	27151 (47.89)	3.993	23003 (46.28)	3784	27563 (55.64)	7.859	35869 (172.98)	7.302
TO	0 (0)	0	202949 (91.43)	52.596	213.19 (94.44)	56.895	513 (0.23)	189	232374 (102.39)	63719	240009 (208.08)	65934
**North**	1037440 (43.67)	288473	1830847 (79.48)	530.303	1938942 (84.85)	547.28	1810481 (76.66)	493698	2004008 (10.55)	569.995	2205757 (97.25)	599.325
AL	339001 (95.83)	129.71	327489 (92.57)	118.273	335106 (94.73)	117.237	344282 (95.27)	123581	338665 (92.02)	136.844	16914 (4.27)	6.265
BA	309535 (16.89)	62787	1250540 (58.82)	247.779	1345255 (63.27)	266.799	1337716 (58.96)	294333	60743 (2.68)	13.897	1529250 (66.04)	341.257
CE	620028 (56.01)	302038	1071220 (94.91)	484.083	1036645 (96.71)	465.517	1139437 (101.78)	513986	1114015 (89.5)	510.456	1186524 (91.88)	513.233
MA	506891 (63.21)	243236	801882 (99.11)	353.789	778586 (93.59)	350.236	807576 (95.89)	353392	781953 (89.93)	345.398	743613 (84.26)	327.132
PB	255951 (49.35)	78983	443432 (85.5)	134.426	467128 (90.07)	139.63	448356 (86.83)	136677	459386 (88.97)	147.101	490633 (93.92)	154.575
PE	69332 (6.43)	28202	302896 (27.89)	103.753	742416 (70.74)	252.156	598212 (52.59)	211916	175882 (15.46)	55.125	561031 (50.23)	203.575
PI	83626 (19.01)	31507	166826 (38.66)	59.115	417.31 (93.25)	150.371	407554 (89.89)	151.42	405348 (86.87)	152.837	424848 (91.02)	162101
RN	83333 (17.84)	31148	405776 (83.42)	136.627	420272 (82.61)	247.678	412137 (80.44)	154775	381785 (71.95)	144.326	446691 (83.6)	176.086
SE	188982 (75.17)	53663	215319 (85.65)	57.272	218865 (86.56)	59.354	219.85 (89.48)	67393	135958 (55.33)	38013	209221 (84.69)	61.818
**North east**	2456679 (35.86)	961274	4985380 (69.32)	1.695.117	5761583 (80.46)	2.048.978	5715120 (76.64)	2007473	3853735 (50.39)	1.543.997	5608725 (72.16)	1.946.042
ES	40.15 (8.21)	8962	32593 (6.67)	6.462	0 (0)	0	51.04 (9.28)	10611	257.51 (41.35)	49296	310196 (49.09)	62.734

MG	350139 (11.89)	50848	2730579 (90.25)	372.34	99268 (2.97)	20818	2928544 (87.48)	425778	2496814 (71.55)	358.689	2488052 (70.74)	378.712
RJ	226479 (11.33)	38625	295053 (14.76)	54.141	834063 (42.82)	162.917	534686 (30.56)	141797	683768 (41.54)	150.791	1274055 (88.71)	325.692
SP	1121637 (21.06)	194757	3818778 (65.03)	772.851	3198583 (54.31)	692.785	367095 (6.74)	68719	3362383 (71.05)	805.51	3653446 (74.5)	914.622
**South East**	1738405 (16.16)	293192	6877003 (60.4)	1.205.794	4131914 (35.21)	876.52	3881365 (34.99)	646905	6800475 (64.82)	1.364.286	7725749 (73.65)	1.681.760
PR	0 (0)	0	103319 (92.43)	14.265	56.78 (75.56)	8.161	25716 (90.23)	2305	0 (0)	0	()	
**South***	0 (0)	0	103529 (92.62)	14265	56.78 (75.56)	8161	25716 (90.23)	2305	0 (0)	0	()	
DF	0 (0)	0	134295 (42.88)	17493	80301 (26.07)	11189	0 (0)	0	0 (0)	0	0 (0)	0
GO	523442 (47.57)	48627	926965 (86.75)	87.844	859.13 (95.64)	77.219	3348 (0.3)	344	975435 (86.85)	99.728	1013008 (90.05)	103.385
MS	297371 (64.14)	55298	316067 (68.17)	61.469	351471 (75.81)	75.134	382964 (82.25)	91.51	153362 (1.05)	29186	247442 (46.66)	49797
MT	101358 (19.06)	19316	403626 (76.1)	86.611	445741 (82.78)	85.457	447692 (81.68)	79731	475157 (85.09)	89.638	489469 (86.53)	93906
**Central-west**	922171 (38.28)	123241	1780953 (74.96)	253.417	1736643 (78.64)	248.999	834004 (34.11)	171585	1603954 (9.64)	218.552	1749919 (69.19)	247088
**Brazil**	6154695 (27.33)	1666180	15577712 (66.66)	3.698.896	13625862 (58.07)	3.729.938	12266686 (52.45)	3321966	14262172 (26.52)	3.696.830	17290150 (74.98)	4.474.215

VC: Vaccination coverage. *RS and SC do not have a rabies prophylaxis program for dogs and cats.
